# View on a mechanistic model of *Chlorella vulgaris* in incubated shake flasks

**DOI:** 10.1007/s00449-021-02627-2

**Published:** 2021-10-22

**Authors:** Fabian Kuhfuß, Veronika Gassenmeier, Sahar Deppe, George Ifrim, Tanja Hernández Rodríguez, Björn Frahm

**Affiliations:** 1 Biotechnology and Bioprocess Engineering, Ostwestfalen-Lippe University of Applied Sciences and Arts, Campusallee 12, Lemgo, Germany; 2 Fraunhofer IOSB, Lemgo, Germany; 3 “Dunarea de Jos” University of Galati, Galati, Romania

**Keywords:** Microalgae, Parameter estimation, Light intensity, Light duration, Radiative model

## Abstract

**Abstract:**

Kinetic growth models are a useful tool for a better understanding of microalgal cultivation and for optimizing cultivation conditions. The evaluation of such models requires experimental data that is laborious to generate in bioreactor settings. The experimental shake flask setting used in this study allows to run 12 experiments at the same time, with 6 individual light intensities and light durations. This way, 54 biomass data sets were generated for the cultivation of the microalgae *Chlorella vulgaris*. To identify the model parameters, a stepwise parameter estimation procedure was applied. First, light-associated model parameters were estimated using additional measurements of local light intensities at differ heights within medium at different biomass concentrations. Next, substrate related model parameters were estimated, using experiments for which biomass and nitrate data were provided. Afterwards, growth-related model parameters were estimated by application of an extensive cross validation procedure.

**Graphic abstract:**

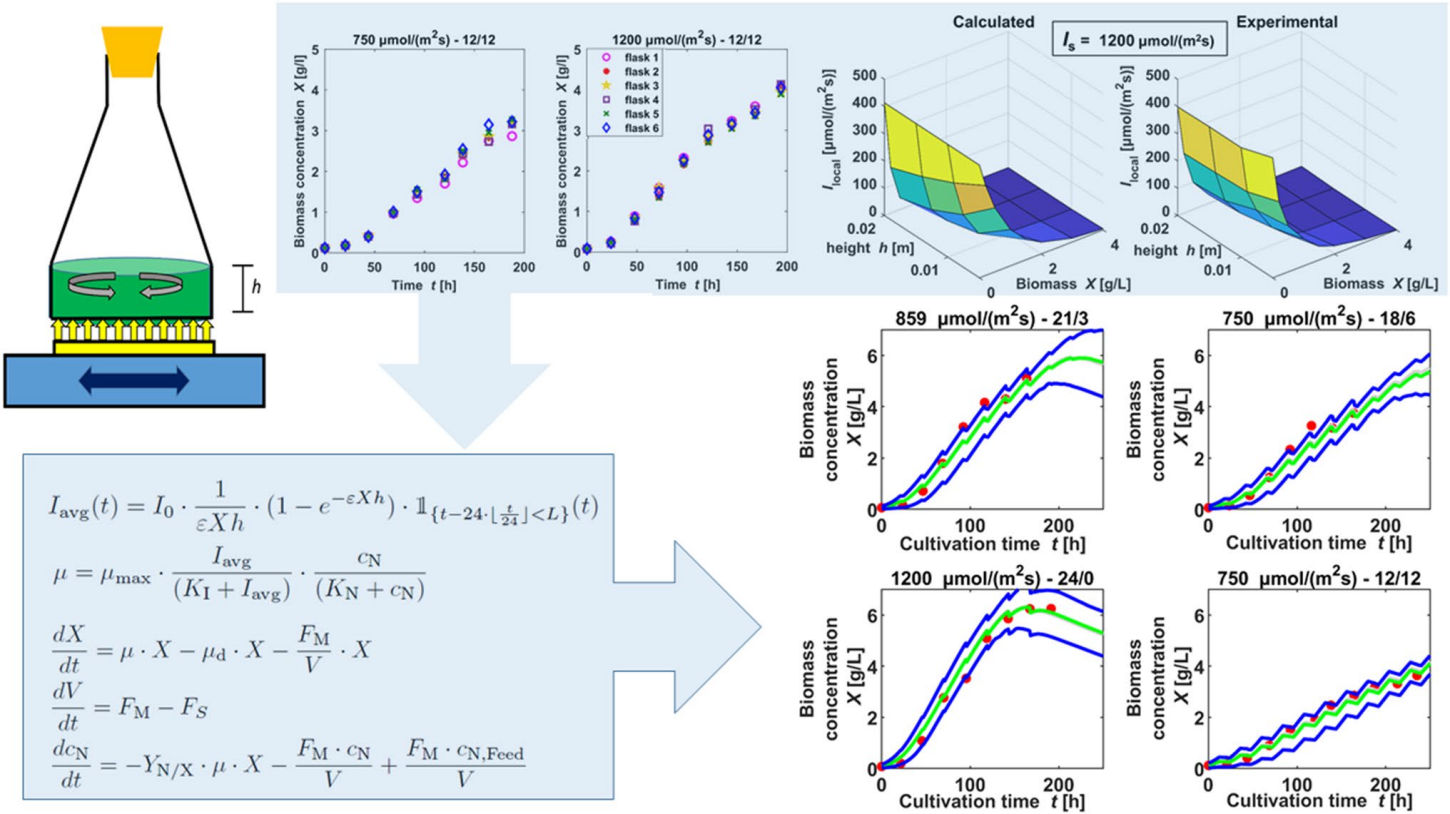

**Supplementary information:**

The online version contains supplementary material available at 10.1007/s00449-021-02627-2.

## Introduction

Microalgae as a quickly growing renewable resource allow the production of biofuels, nutraceuticals, cosmetics, and pharmaceuticals [1, 2]. In order to produce a sufficient amount of biomass, it is necessary to know how the microalgae will grow under the chosen cultivation conditions.

Since microalgae produce energy photosynthetically, light is the primary substrate which is attenuated with increasing cell density [3, 4]. This is due to scattering, shading, and absorption by the cells. Additionally, exhaustion of macro- and micronutrients, accumulation of oxygen and low availability of carbon-dioxide can inhibit biomass production [5]. Moreover, parameters like temperature, lighting duration, pH-value, and agitation have to be taken into account for the optimization of the cultivation of microalgae [6]. To describe such a cultivation process, and to gain better understanding, it is beneficial to provide a mathematical model.

Various models for describing the growth kinetics of phototrophic microalgae have been developed over the past decades. Most recent models are based on Monod kinetics, an example is the Tamiya model, which uses *Chlorella ellipsoidea* as test organism and describes the specific growth rate in microalgae cultures with light as the limiting substrate in flat flasks [7]. This approach is also followed in a publication of Yun et al. [8]. An important milestone for the modeling of algae is the work of Cornet et al. [9], in which the kinetics and energetics of photosynthetic organisms in various setups (e.g. different photobioreactors, feeding strategies, biomass concentrations) were researched, which resulted in the mathematical description of different scenarios. The model of Filali et al. [10] uses a combination of light intensity based on Monod kinetics with light limitation and the total inorganic carbon dioxide concentration (TIC) for each cell in the culture. Similar studies are described in the publication by Concas et al. [11]. Here the photosynthetically produced oxygen and pH value were also factored in. Other examples of algae modeling are described by He et al. [12] and Mairet et al. [13]. The model of Aslan and Kapdan [14] is also based on Monod kinetics but refers to light, nitrogen (ammonium), and phosphorus (phosphate) as limiting factors. A similar approach has been taken by Eze et al. [15], which consider light, carbon, nitrogen, and phosphate as limiting substrates in a Monod kinetic model.

Another important factor for growth is the temperature, which is assumed by most models to be constant. Béchet et al. [16] use light and temperature in combination as the variables for their modeling process. In Ifrim et al. [17] a dynamic pH model is presented, which is able to predict the pH-value based on the chosen cultivation strategy. Hereby through the definition of multiple mass balances (e.g. dilution rate, light intensity, temperature, gas flow rates) the change of the system pH and its influence on the growth can be calculated and displayed.

The above-mentioned modeling approaches describe the growth of various microalgae in various cultivation systems (e.g., photobioreactors, polyethylene bottles) and process definitions (constant and varying factors), but rarely for shake flask cultivation. This laboratory-scale gives the opportunity to develop an optimal process strategy, perform screening tests, and save time and costs because of the capability of multiple parallel experiments. The setting of 12 flasks with illumination from the bottom allows variation of light intensity and light duration in wide ranges.

In this contribution, first, a mechanistic model was adapted from existing bioreactor models, and attuned to the microalgae *C. vulgaris*, cultivated at laboratory-scale in shake flasks, with illumination from the bottom. Some recent photobioreactor models for *C. vulgaris* consider light duration as a set cultivation factor, but it is fixed at 12 or 18 h per day [11, 18, 19] and cannot vary in wide ranges. Lee et al. [20] suggest to focus on a better mathematical expression of light, concerning light attenuation and also temporal variation of light intensity. The shake flask setting allows to generate multiple data sets with different light intensities and light durations. So in the next step, nine different settings with several parallel cultivations were conducted, that supplied a broad data base for parameter estimation and validation.

In a third step, parameter estimation was carried out. The parameters concerning light were determined using additional measured results for local light intensities. Substrate related parameters were estimated on cultivation data with supplementary nitrate measurements. For the estimation of growth parameters the various cultivation data were used. A Leave-One-Out cross validation was performed to evaluate, if simulations can predict biomass growth for diverse light settings.

## Materials and methods

### Cultivation conditions

#### Microorganism and growth medium

The microalgae *C. vulgaris* was obtained from the Collection of Algae Cultures of the University of Göttingen (SAG, Germany). The media used for maintenance and experimental studies on *C. vulgaris* was a synthetic modified Kessler medium according to suggestions of Mandalam and Palsson [21], the composition is given in Table 11 of the Online Resource. After autoclavation, a pH of 6.3 was obtained, which does not need to be adjusted further due to the included buffer system, as stated by Kessler [22].

#### Experimental setup

A custom LED lighting tablet (Almostec GmbH, Austria), specially constructed for the cultivation of microalgae in shake flasks, was used in these experiments. The lighting tablet allows the cultivation of twelve 500 mL flasks in parallel. In order to minimize the distance between the light source and the cultures, the LEDs are placed directly beneath the shake flasks. The heat output of the LEDs is low even at high light intensities. The use of three external power supply units (GPS-2303, GW Instek, Taiwan) made it possible to regulate the photosynthetically active radiation (PAR [$\upmu$mol /(m$^2$ s)]) of six pairs of shake flasks independently. The PAR attained from the specialized light source ranged from 57 to 1200 $\upmu$mol /(m$^2$ s). The lighting tablet was mounted on top of an orbital shaker (MaxQ2000CO2, Thermo Fisher Scientific Inc., USA), which was placed inside an incubator (ICOmed240, Memmert GmbH, Germany), allowing the regulation of carbon dioxide concentration in the culture atmosphere as well as the relative humidity. To dissipate the waste heat of the shaker, cooling was applied: The thermostat of the incubator with the external cooling thermostat (FRIGOMIX R 2000, Sartorius) and a radiator (Phobya Xtreme) kept the temperature constant at 25 $^{\circ }$C.

#### Cultivation conditions

Cultures were incubated at 25 ± 1 $^{\circ }$C under a 5 % CO$_2$ atmosphere to provide optimal conditions for microalgal growth. The orbital shaker was set to 100 rpm (orbit diameter: 19 mm) to allow for homogenous mixing and lighting conditions in the culture medium. Cultivation took place in 500 mL narrow neck, unbaffled, glass conical flasks (Schott Duran, Germany). Biomass concentration was determined in the precultures and inoculated into 200 mL of Kessler medium. Concentration at the start of cultivation was 0.1 g/L for each experiment.

### Experimental data

**Fig. 1:**
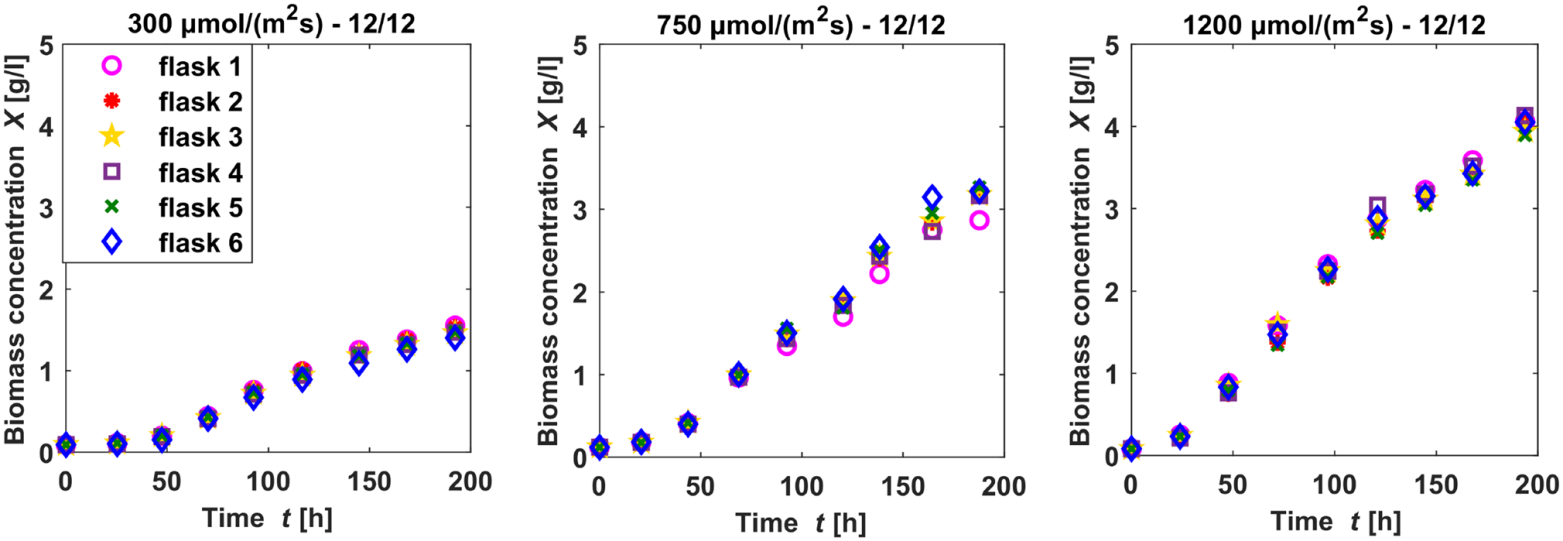
Comparison of low (left, 300 $\upmu$mol /(m$^2$ s)), medium (middle, 750 $\upmu$mol /(m$^2$ s)) and high (right, 1200 $\upmu$mol /(m$^2$ s)) light intensities, all at a light duration of 12 h per day. All experiments were performed in groups of six shake flasks

To provide experimental data for parameter estimation and model validation, the 12 places on the light panel were used to generate multiple data sets: 12 parallel cultivations at the same light intensity and duration were performed, as well as cultivations in nine different light intensities or durations. In two of the experiments, nitrate concentrations were measured to supply data concerning nitrate uptake. Table 1 gives an overview on the 54 different datasets generated: Experimental data were taken once per day, whereas a minimum of 180 h of cultivation was performed. Experiments were done with 6–12 shake flasks at a time, sometimes using the possibility to apply different light intensities for each pair of two. Identical shake flasks were used to carry out all experiments, while these were randomly assigned to a lighting area for each application. An exemplary set of cultivation data is shown in Fig. 1.

**Table 1:** Light conditions and number of cultivations

Dataset no.	Light conditions: Intensity [$\upmu$mol/(m$^2$ s)]	Light–dark [h]	Number of parallel cultivations
# 1	536	12–12	6
# 2	1200	12–12	6
# 3	300	12–12	6
# 4	859	18–6	2
# 5	859	21–3	4
# 6	750	18–6	6
# 7	750	24–0	2
# 8	1200	24–0	12
# 9	750	12–12	12

### Analytics

#### Sampling

Sampling comprehended taking 7.5 mL of culture liquid under sterile conditions and pipetting back the same amount of fresh culture medium into the culture flasks. This proceeding was chosen to maintain a constant working volume in the flasks although about ten samples were taken out of each. A constant working volume is necessary to keep mixing conditions and distribution of photons in the flasks constant during cultivation.

#### Bio dry mass concentration [g/L] via optical density (OD)

To determine bio-mass concentration, a correlation of dry mass concentration and the optical density of the culture broth was prepared. The correlation equation was determined via linear regression out of seven different concentrations with $R ^{2}$ = 0.99. The optical density ($\lambda$ = 740 nm) was determined in a spectrophotometer (DR 3900, Hach Lange GmbH, Germany). This wavelength was chosen since it lies outside of the absorption spectrum of the pigments (chlorophylls, carotenoids) in *C. vulgaris* and therefore allows for a more accurate correlation ([23]). All experiments were done in triplets.

#### Chlorophyll and carotenoids

During the conduction of the experiments the concentrations of chlorophyll and carotenoids were measured in order to check on the status of *C. vulgaris* and detect cell damage or a possible lack of nutrients while cultivating. No bleaching effect due to intense lighting of the cells was found during the research. The extraction of chlorophylls a and b and carotenoids was executed according to the instructions of [24] and [25]. All experiments were done in triplets.

#### pCO$_{2}$ and pO$_{2}$

For the measurement of the dissolved CO$_{2}$ (pCO$_{2}$), the measuring probe InPro5000® CO$_{2}$ Sensor (Mettler Toledo, USA) and the M400 process transmitter (Mettler Toledo, USA) were used. For the measurement of the dissolved O$_{2}$ (pO$_{2}$), the measuring probe OxyFermTM FDA VP 225 (Hamilton Company, USA) was applied. To calibrate the measuring probe, the bioreactor control software “medorex bioreactor” (medorex e.K., Germany) was used.

#### pH values

The pH values were monitored in each sample with a pH-meter (ph521, WTW, Weilheim).

#### Nitrogen/nitrate-concentrations

The measurement of nitrate concentration in the samples was performed using the method from [26].

#### Incident light intensity measuring

Light measurements were performed using the Universal Light Meter “ULM-500” from Heinz Walz GmbH, which was equipped with Spherical Micro Quantum Sensor US-SQS/L. The device gives the total light of the visible spectrum in photosynthetically active radiation (PAR) [$\upmu$mol /(m$^2$ s)]. PAR describes the solar radiation in the wavelength spectrum of 400–700 nm, which is the relevant spectrum for the photosynthetic activity of plants. Hereby PAR is defined by the difference between energy-based PAR (W/m$^{2}$) (energy of the photons per area) and photon-based PAR (PPF (photosynthetic photon flux, (photons per time) and PFD (photon flux density) or PPFD (photosynthetically active photon flux density, amount of photons within 400–700 nm per area and time)) in [$\upmu$mol /(m$^2$ s)]. The chosen light meter hereby measures and calculates PAR as the light intensity in PPFD, which is the more common method of measurement. The measurements were used to adjust the parameters in the equation of the radiative model. Therefore, the attenuated light intensity was measured in Kessler medium without algae as well as in algae suspension with different dry mass concentrations and in different heights above the bottom of the flasks inside the fluid, to explore the light attenuation due to the growing algae.

### Estimation of model parameters

Parameter estimation has been performed with the Nelder-Mead simplex algorithm [27], using MATLAB to solve the optimization problem with the goal of minimizing an objective function, which describes the discrepancy between measured and simulated data ([28]). In this work the sum of the weighted squares of the differences between simulated and experimental values divided by the maximum value of the corresponding data set is used in the objective function. The experimental data were divided in training data and validation data for each estimation, leaving out one data set of every setting for final tests. The exact attribution of the data sets is given in Tables 15 and 18 in the Online Resource. Parameter estimation was performed 100 times, sampling the initial concentrations out of a normal distribution with 3% relative standard deviation in each iteration to account for measurement errors.

## Results and discussion

The following chapter first shows the steps and assumptions taken for the setup of the model and then describes parameter estimation. Finally the simulations are compared to experimental datasets assessing the goodness of fit.

### Structure of the photosynthetic growth model

#### The radiative model

To determine the incident light intensity $I_\mathrm{0}$, light was measured in the pure medium within the fluid in the flasks. Thus, the fraction of $I_\mathrm{S}$ (set light intensity of the light source, here: lighting tablet) that actually arrives inside the flasks, i.e. $I_\mathrm{0}$, was found to be described by the following regression equation:

1 $$\begin{aligned} \begin{aligned}&I_\mathrm{0}&= 6.92 + 0.436 \cdot I_\mathrm{S} - 8.1 \cdot 10^{-5} \cdot I_\mathrm{S}^2 \end{aligned} \end{aligned}$$

**Fig. 2:**
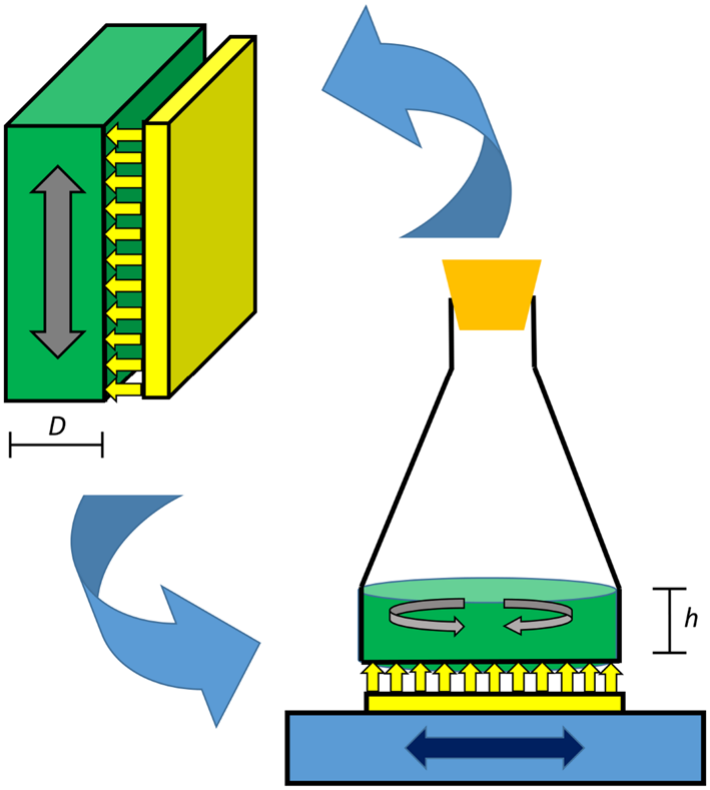
Schematic diagram of the geometric conditions in the lighted and shaken flasks compared with a flat bioreactor setting; considering radiation, the width of the bioreactor *D* is equivalent to the height of the fluid in the flask *h*

The setting for one flask above the light source on the shaking tablet is displayed in Fig. 2. The geometric conditions of a shaken flask illuminated from the ground are similar to those of a flat bioreactor that is illuminated from one side. The radiation beam length *D*, which is the width of the bioreactor, corresponds to the radiation beam length *h*, which is the height of the working volume in the flask. As stated in [9] for bioreactors in general, the light intensity at any given point inside the culture can be described by applying the Lambert-Beer equation. This equation can also be applied in the shake flasks, since the light path through the culture is analogous, see Fig. 2.

2 $$\begin{aligned} \begin{aligned}&I&= I_\mathrm{0} \cdot e^{-(\varepsilon \cdot X \cdot z)} \end{aligned} \end{aligned}$$

*I* describes the local light intensity [$\upmu$mol /(m$^2$ s)] subject to the optical path length *z* [m] and the biomass concentration *X* [g/L]. The coefficient $\varvec{\varepsilon }$ [m$^{2}$/kg], Eq. 3, has a constant value, due to the assumption of complete homogeneity of the system: $\alpha$, the linear scattering modulus (dimensionless), as described by J. F. Cornet [29], is constant inside the fluid. Parameter $\alpha$ derives from the mass absorption coefficient $E_\mathrm{a}$ [m$^{2}$/kg], the mass scattering coefficient $E_\mathrm{s}$ [m$^{2}$/kg] of the microalgae and *b*, the dimensionless backward scattering fraction.

3 $$\begin{aligned} \begin{aligned} \varepsilon&=\dfrac{1+\alpha }{2\alpha }\cdot E_\mathrm{a} &\alpha&=\sqrt{\dfrac{E_\mathrm{a}}{E_\mathrm{a}+2b\cdot E_\mathrm{s}}} \end{aligned} \end{aligned}$$

In order to set up a radiative model describing the light intensity over the entire system, the averaged light intensity $I_\mathrm{avg}$ is calculated, based on the following assumptions: Since the shake flask is constantly in motion via the orbital shaker, it can be assumed that there is complete homogeneity in the system, which would mean that an individual algae cell can be found at any point in the system with the same frequency (well mixed) and that the biomass will be exposed to an average light intensity in the whole volume. Thus $I_\mathrm{avg}$ can be computed by the volume integral of the system. Further explanations to this calculation can be found in the second section in the Online Resource.

4 $$\begin{aligned} \begin{aligned} I_\mathrm{avg}&=\dfrac{1}{V}\cdot \int _{}^{}\int _{}^{}\int _{V}^{}I_0 \cdot e^{-(\varepsilon \cdot X \cdot z)}dV \end{aligned} \end{aligned}$$

As the lighting in the investigated setting is located under the flask in upright direction and evenly distributed over the whole surface, it can be assumed that the incident light is equal for all cells in the same level above the ground. So, the incident light on the shaken cells in the flask only depends on *z*, leading to

5 $$\begin{aligned} \begin{aligned} I_\mathrm{avg}&=\dfrac{1}{V}\cdot \int _{0}^{h}I_0 \cdot e^{-(\varepsilon \cdot X \cdot z)}\,dz\,=\,I_0\cdot \dfrac{1}{h\cdot \varepsilon \cdot X}\cdot (1-e^{-(\varepsilon \cdot X \cdot h)}) \end{aligned} \end{aligned}$$

$\varepsilon$ is calculated according to Eq. (3) out of the light parameters $E_\mathrm{a}$, $E_\mathrm{s}$ and *b*. The radiative model equation describes the light attenuation inside the culture, that occurs by the growing biomass during cultivation.

To describe the implication of light duration as the second independent variable, a function for light duration was needed. The light duration terms in literature only model day–night cycles with little alterations, whereas in the setting described here, light intensity and duration are adjustable at will. So, a new term for the use of artificial light in any desired duration was added: in the lighting beneath a flask the light is either on or off, having thus the shape of a pulse signal. The case is mathematically described as following:

6 $$\begin{aligned} I_\mathrm{avg}(t) = I_\mathrm{0} \cdot \frac{1}{\varepsilon X h} \cdot (1-e^{-\varepsilon X h}) \cdot \mathbbm {1}_{ \lbrace t-24\cdot \lfloor \frac{t}{24} \rfloor < L \rbrace } (t) \end{aligned}$$

with indicator function $\mathbbm {1}_{\lbrace t-24\cdot \lfloor \frac{t}{24} \rfloor < L \rbrace }$, that is possessing either value 1 (if $t-24\cdot \lfloor \frac{t}{24} \rfloor < L$) or value 0 (else). The variable *L* stands for hours of lighting per day [h].

#### Kinetic equation

The main equation to describe the growth of *C. vulgaris* can be categorized as describing the conversion of light and nutrients, such as nitrate, into biomass. According to [20], light and nitrogen concentration (here: nitrate) were found to be limiting substrates. During several experiments under the chosen cultivation conditions, light limitation was always observed, whereas the nitrate concentrations were in most cases high enough to prevent limitation. To account for light and nitrate limitation, the commonly used Monod [30] equation was applied:

7 $$\begin{aligned} \begin{aligned} \mu&=\mu _\mathrm{max}\cdot \dfrac{I_\mathrm{avg}}{(K_\mathrm{I}+I_\mathrm{avg})}\cdot \dfrac{c_\mathrm{N}}{(K_\mathrm{N}+c_\mathrm{N} )} \end{aligned} \end{aligned}$$

In this equation $\mu$ [1/h] is the actual growth rate, $\mu _\mathrm{max}$ [1/h] is the specific maximum growth rate. The parameter $K_\mathrm{I}$ [$\upmu$mol /(m$^2$ s)] displays the half saturation constant for light, $K_\mathrm{N}$ [g/L] the half saturation constant for nitrate and $c_\mathrm{N}$ [g/L] is the nitrate concentration.

#### pH-influence

Regarding the published literature on the cultivation of *C. vulgaris*, it is known that processes with this alga are rarely pH controlled. As referred in the publication of Kong et al. [31], during photoautotrophic growth the pH-value rises. In the performed experiments the pH rose according to growth, to a maximum pH of 8.5 at 1200 $\upmu$mol /(m$^2$ s) and 24 h of light daily. Examples for pH-values are given in Fig. 10 in the Online Resource. This behavior can be explained by the buffer substances NaH$_{2}$PO$_{4} \cdot$ 2 H$_{2}$O and Na$_{2}$HPO$_{4} \cdot$ 12 H$_{2}$O contained in the media that buffer the pH in a range of pH 6–8. At various combinations of light intensity and light durations no significant influence of the pH on the growth of the algae could be stated. These results agree with the results of Gong et al. [32], who examined the influence of light and pH on the cell density of *C. vulgaris* in a light incubator. Strategies with and without pH control were compared to each other and the authors stated that no significant difference of the obtained cell densities of both strategies could be found. So it can be assumed that the pH in the observed ranges has no significant influence on growth, therefore it was not further considered in the model.

#### Mass balance

To model biomass growth, volume changes and nitrate uptake, the following main differential equations were formulated:

8a $$\begin{aligned}&\frac{dX}{dt} = \mu \cdot X - \mu _\mathrm{d} \cdot X - \frac{F_\mathrm{M}}{V} \cdot X \end{aligned}$$

8b $$\begin{aligned}&\frac{dV}{dt} = F_\mathrm{M} - F_S \end{aligned}$$

8c $$\begin{aligned}&\frac{dc_\mathrm{N}}{dt} = - Y_\mathrm{N/X} \cdot \mu \cdot X - \frac{F_\mathrm{M}\cdot c_\mathrm{N}}{V} + \frac{F_\mathrm{M} \cdot c_\mathrm{N,Feed}}{V} \end{aligned}$$

The time course of biomass concentration *X* [g/L] is determined by the actual growth rate $\mu$ [1/h], the specific death rate $\mu _\mathrm{d}$ [1/h] and dilution $F_\mathrm{M}/V$, when fresh medium is added.

The volume change is the difference between the feed rate $F_\mathrm{M}$ [L/h] and the sampling rate $F_\mathrm{S}$ [L/h]. The feed rate $F_\mathrm{M}$ is hereby defined as the amount of medium volume that is added to the system over time, whereas the sampling rate $F_\mathrm{S}$ [L/h] describes the amount of volume which is removed from the system over time (here: due to sampling). In our case the sampling rate $F_\mathrm{S}$ [L/h] is equal to the medium feed rate $F_\mathrm{M}$ [L/h], so that the working volume *V* [L] is constant. Although this equation is set to a constant value here, it would allow for the adaption to a fed-batch strategy or to add medium at a different point in time than sample taking.

The change of the nitrate concentration over time is modeled in Eq. 8c. Nitrate concentration decreases according to the rate of nitrate uptake of the present algae. This uptake depends on the metabolic activity of the algae, dependent upon $\mu$ [1/h], and on their concentration, *X *[g/L]. To account for this, a model was chosen according to the equation used by Del Rio et al. in ([33]). $Y_\mathrm{N/X}$ [$\mathrm g_\mathrm{nitrate}/\mathrm g_{biomass}$] is the nitrate yield coefficient and $K _\mathrm{N}$ [mg/L] as nitrate Monod constant is included in $\mu$ in Eq. (7). The nitrate concentration increases when the sample volume is replaced by fresh media, depending on the difference between the actual concentration $c_\mathrm{N}$ [g nitrate/L] and that of the fresh media $c_\mathrm{N,Feed}$ [g nitrate/L] (Table 2).

**Table 2:** Full list of variables and parameters in the model equations

Parameter	Definition	Unit
*X*	Biomass concentration	(g/L)
*t*	Time	h
$\mu$	Specific growth rate	1/h
$\mu _D$	Specific death rate	1/h
$\mu _\mathrm{max}$	Maximum specific growth rate	1/h
$F_\mathrm{M}$	Medium feed rate	L/h
$F_\mathrm{S}$	Sample rate	L/h
*V*	Volume	L
$I_\mathrm{S}$	Set Light intensity of the light source	μmol/(m^2^s)
$I_\mathrm{0}$	Incident light intensity	μmol/(m^2^s)
*L*	Light duration	h
$\mathbbm {1}_{\lbrace t-24\cdot \lfloor \frac{t}{24} \rfloor < L \rbrace }$	Indicator Function to switch light on or off	
$I_\mathrm{local}$	Local light intensity	μmol/(m^2^s)
*z*	Height of a specific localization in the flask	m
*h*	Height of liquid in the flask	m
$I_\mathrm{avg}$	Average light intensity	μmol/(m^2^s)
$\varepsilon$	Ratio spherical to hemispherical light per biomass concentration and height	m^2^/kg
$\alpha$	Linear scattering modulus	–
$E_\mathrm{a}$	Mass absorption coefficient	m^2^/kg
$E_\mathrm{s}$	Mass scattering coefficient	m^2^/kg
*b*	Backward scattering fraction	–
$K_\mathrm{I}$	Light associated Monod kinetic constant	μmol/(m^2^s)
$K_\mathrm{N}$	Nitrate uptake rate associated Monod kinetic constant	g/L
$c_\mathrm{N}$	Nitrate concentration	g/L
$c_\mathrm{N,Feed}$	Nitrate concentration in the Feed	g/L
$Y_\mathrm{N/X}$	Ratio of maximum nitrate uptake rate and maximum specific growth rate	$\mathrm g_\mathrm{nitrate}/\mathrm g_{biomass}$

### Parameter estimation

The variety of the experimental data provided good conditions for parameter estimation. A wide range of settings, concerning light intensity and duration was represented by data sets, which comprised of at least 2, but mostly 6 or more different cultivations, as specified in Table 1. So it was possible to choose a different data set for each setting as training data, validation data and test data. Which data sets were used respectively, is given in Tables 15 and 18 in the Online Resource.

An adequate starting value is needed for every parameter to be estimated. In order to fix reasonable bounds in the estimation, it is necessary to know in what ranges every parameter is located. Since the parameters found in literature are not necessarily transferable to a setting of *C. vulgaris* in shake flasks, starting values and ranges were explored with additional experiments: Nitrate was measured in a setting with nitrate limitation and the incident light was measured at different heights and algae concentrations. The initial values and the bounds for the three parameter estimations are based on these experimental data, along with parameters from literature, as described in the specific section for each estimation. As the light attenuation and substrate limitation both affect the growth rate, the parameters concerning light interfere with those concerning nitrate uptake. To get the best benefit from the information in the data sets, the model parameters in this work were identified by applying a stepwise parameter estimation. That means that before the parameter estimation using the biomass data of all the cultivations, the light data was used to explore the parameters of $E_\mathrm{a}$, $E_\mathrm{s}$ and *b*, and the nitrate data from several cultivations was used to explore the parameters $K_\mathrm{N}$ and $Y_\mathrm{N/X}$.

#### Parameter estimation for local light intensity

**Fig. 3:**
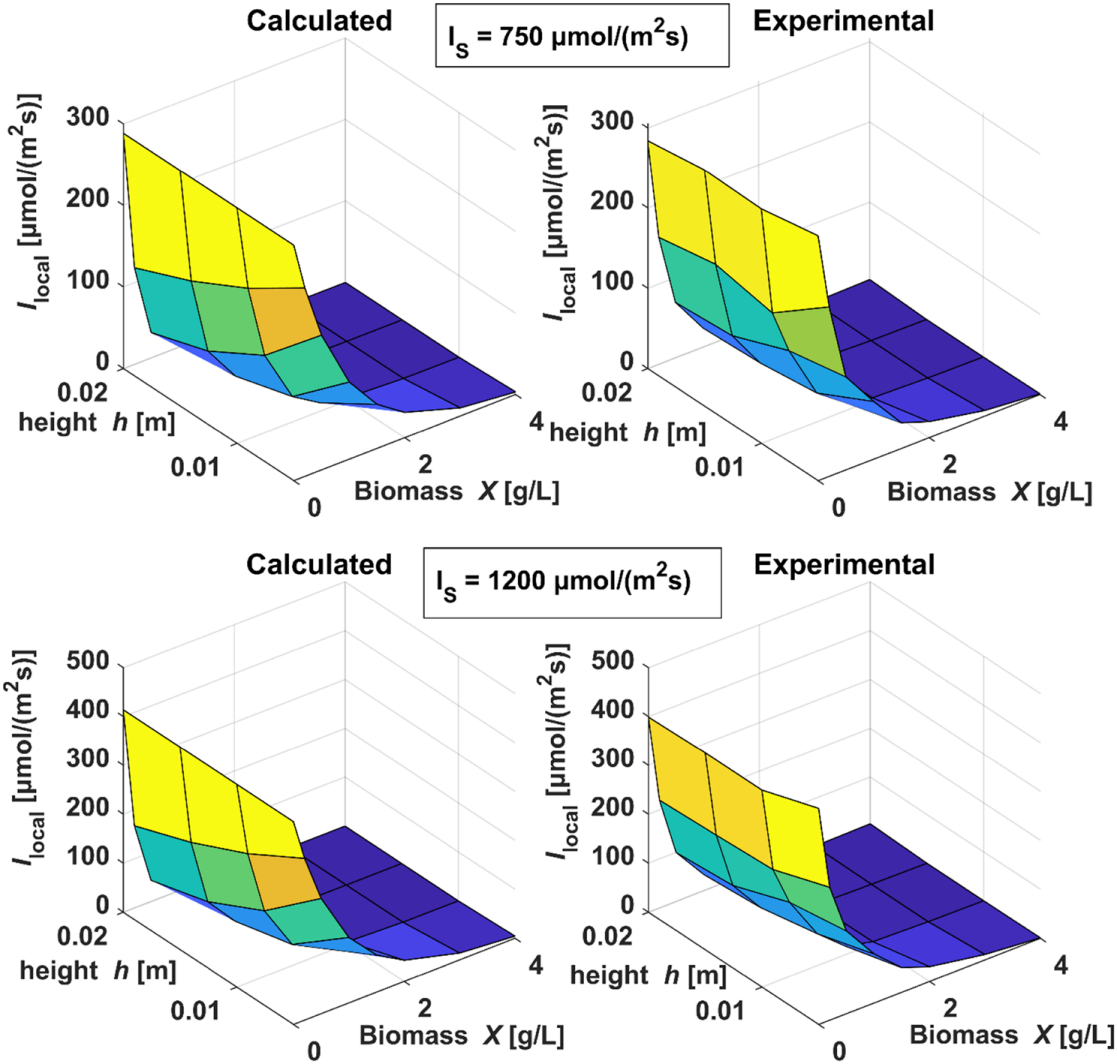
Comparison of calculated local light intensities $I_\mathrm{local}$ with local light intensities measured at different heights (*h*) and biomass concentrations (*X*)

In order to identify the values of $E_\mathrm{a}$, $E_\mathrm{s}$ and *b* in the flask setup, light measurements in flasks with different biomass concentrations and at different heights in the flasks were conducted, Table 12 in the Online Resource states the illuminations, the height values and the biomass concentrations investigated in the light measurements. These data were used in parameter estimations fitting the radiative model Eqs. (5) and (3) to the measured light values. In literature the values for the parameters of the light equation derive from differing experimental setups [34, 35]. Here, as initial values of $E_\mathrm{s}$, $E_\mathrm{a}$ and *b*, those from Pottier et al. [34] were adopted as listed in Table 3. Parameters were estimated without bounds for each illumination value $I_\mathrm{S}$, and for all illumination values together. Table 3 provides the estimated parameters next to the initial values. An example for the comparison of the calculated (simulated) and experimental light data applying the estimated parameters is illustrated in Fig. 3.

**Table 3:** Displayed are the individual parameter estimation results of single data sets for each light intensity and the results of the parameter estimation with all data sets combined

Estimated parameter		Initial value	300 $\upmu$mol/(m$^2$s)	750 $\upmu$mol/(m$^2$s)	1200 $\upmu$mol/(m$^2$s)	Data combined
$E_\mathrm{a}$	[m$^{2}$/kg]	172	243	224	227	227
$E_\mathrm{s}$	[m$^{2}$/kg]	870	756	812	801	801
*b*	[–]	0.0008	0.0007	0.0008	0.0008	0.0008

The estimated parameter $E_\mathrm{a}$ ranges from 224 to 243 m$^{2}$/kg, $E_\mathrm{s}$ varies between 756 and 812 m$^{2}$/kg and *b* between 0.0007 and 0.0008. The obtained values of these parameters are in a magnitude range that is coherent with their physical meaning. They are consistent to the values given by Pottier et al. ( [34]), who stated ranges from 20 to 400 m$^{2}$/kg for $E_\mathrm{a}$, 700 to 1000 m$^{2}$/kg for $E_\mathrm{s}$ and 0.005 to 0.02 for *b*, depending on wavelength.

The calculated results are very close to the experimental results of incident light, as depicted in Fig. 3 for 750 and 1200 $\upmu$mol /(m$^2$ s). The estimated parameters were also quantified by the coefficient of determination ($R^2$): The outcomes regarding light source intensities of 300, 750, and 1200 $\upmu$mol /(m$^2$ s) are 0.95, 0.96 and 0.96, respectively.

Regarding the results of the parameter estimation shown in Table 3, the estimated values of $E_\mathrm{a}$, $E_\mathrm{s}$ and *b* can all be found within a small range. Therefore, they were set on fixed values in the following parameter estimations. The value of $E_\mathrm{a}$ was set to 227 m$^{2}$/kg, the value of $E_\mathrm{s}$ to 800 m$^{2}$/kg and that of *b* to 0.0008.

#### Parameter estimation for nitrate consumption

For the estimation of the parameters concerning nitrate uptake, there were data sets of six cultivations available that comprised measuring of biomass and nitrate concentration, at two different light intensities. The parameters $\mu _\mathrm{max}$, $\mu _\mathrm{d}$ and $K_\mathrm{I}$ were estimated, along with parameters $K_\mathrm{N}$ and $Y_\mathrm{N/X}$. The starting value for $\mu _\mathrm{max}$ was adopted from Filali et al. [10] and confirmed by values of $\mu$ measured experimentally. The lower and upper bounds were set at 0.02 and 0.16, to assure a reasonable scale. For $\mu _\mathrm{d}$, starting value and bounds were kept in a small range between 0.0005 and 0.002 because higher values of $\mu _\mathrm{d}$ are counterbalanced with higher values of $\mu _\mathrm{max}$ in the parameter estimation. As in the timespan of the cultivations growth was always much higher than death, identifiability of $\mu _\mathrm{d}$ by the data was not adequate for wider bounds. The starting value of 64 $\upmu$mol /(m$^2$ s) for $K_\mathrm{I}$ was adapted from Sasi et al. [36] and the bounds were set considering the measured values for growth rate and light intensities: The highest value of $\mu$ in the experimental data was measured at a local light intensity of about 200 $\upmu$mol /(m$^2$ s), accounting for a half-saturation value of about 100 $\upmu$mol /(m$^2$ s). For the latter parameters, concerning nitrate uptake, the lower and upper bounds were set to a broader range, to allow adaption to the measured nitrate data. For $K_\mathrm{N}$, a value of 0.14 mg/L is given in [14], which was assumed as starting value. The starting value of 0.15 mg/mg for $Y_\mathrm{N/X}$ was derived from the quotient of measured values of nitrate uptake and growth rate in cultivations: Mean value for nitrate uptake divided by growth rate was 0.17 for $I_\mathrm{S}$ = 1200 $\upmu$mol /(m$^2$ s) and 0.14 for $I_\mathrm{S}$ = 536 $\upmu$mol /(m$^2$ s).

All starting values along with the bounds for estimation are given in Table 13 in the Online Resource. The six data sets were alternately divided in four training data sets and one validation data set, leaving out one data set completely for final test. See Table 15 in the Online Resource for the assignment of the data sets. So five runs of parameter estimation were performed giving each a distribution of parameter values, and each run was validated with the dataset not used for training. Table 4 gives the coefficient of determination of simulated values versus validation dataset for each validation and for the test data set:

**Table 4:** Coefficient of determination $R^2$ of simulated and experimental biomass concentration for five data sets and test data in the estimation including nitrate data

Nitrate estimation	
Validation run number	Coefficient of determination $R^2$
1	0.889
2	0.886
3	0.803
4	0.955
5	0.892
Test data	0.949

Each of all five runs gave a resulting parameter distribution that provided a set of five mean parameters. The mean parameter sets from all runs were taken to compute one mean value for each parameter, the resulting parameter values are displayed in Table 5.

**Table 5:** Mean values for parameters estimated with five data sets including biomass and nitrate measuring

Parameter		Mean value
$\mu _\mathrm{max}$	[1/h]	0.135
$\mu _\mathrm{d}$	[1/h]	0.002
$K_\mathrm{N}$	[mg/L]	0.149
$K_\mathrm{I}$	[μmol/(m^2^ s)]	96
$Y_\mathrm{N/X}$	[mg/mg]	0.245

**Fig. 4:**
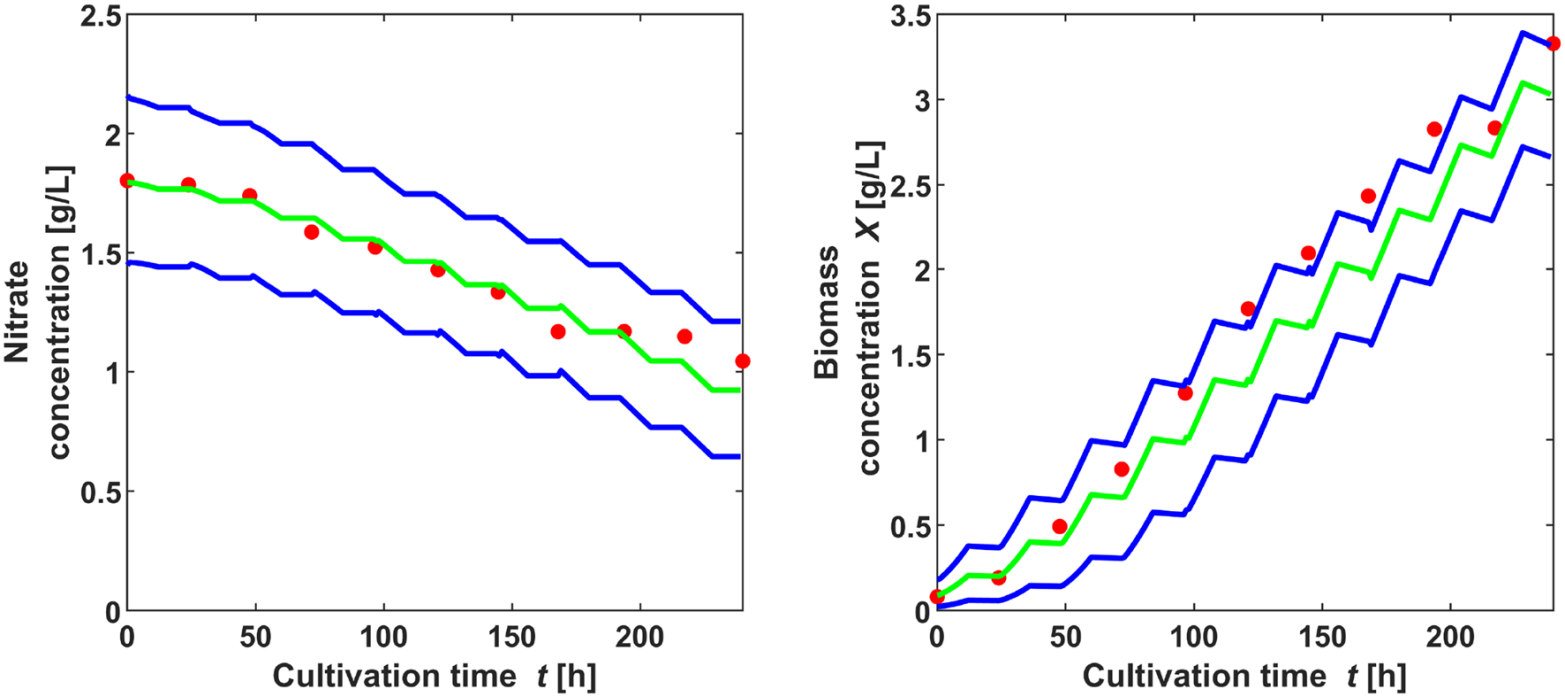
Comparison of calculated nitrate and biomass concentrations (lines) and experimental test data set (dots), applying sampled parameter sets from five estimations (dark lines: 5%- and 95%-quantiles simulation, pale lines: mean simulation)

The test data that had been left over, were evaluated conducting a hundred simulations with a randomly chosen parameter set from the estimations, variating the initial values randomly out of a normal distribution with 3% relative standard deviation. A comparison of the test data set and the simulations of nitrate and biomass with the estimated parameter values is shown in Fig. 4: The red dots show the experimental data not used in the parameter estimation and the lines give the 90% prediction bands of the simulated data. The test data derived from a cultivation at lighting of 536 $\upmu$mol /(m$^2$ s) and 12 h per day.

#### Parameter estimation for the entire model

As the parameters concerning light were well-established in the light estimation, these were no more identified in the main parameter estimation. The values of parameters $E_\mathrm{s}$ and *b* were set to 800 m$^2$/kg and 0.0008, $E_\mathrm{a}$ was set to 227 m$^2$/kg. Likewise, the parameters concerning nitrate uptake were identified in the nitrate estimation and thus $K_\mathrm{N}$ was set to 0.15 g/L and $Y_\mathrm{N/X}$ to 0.24 mg/mg. In the main parameter estimation, three parameters, $\mu _\mathrm{max}$, $\mu _\mathrm{d}$ and $K_\mathrm{I}$ were estimated. For $\mu _\mathrm{max}$ and $\mu _\mathrm{d}$ , the estimated mean values from the nitrate estimation were taken as starting values. Starting value of $K_\mathrm{I}$ was the same as in the nitrate estimation. The bounds for all free parameters were set at +/- 50%. See Table 16 in the Online Resource for all values.

The parameter estimation was performed with the data sets from 9 cultivations of different light settings, as listed in Table 1. The high number of cultivation data sets available permitted a broad variation of training data, validation and test data: Each run used 9 different flasks from the 9 light settings. So the 54 data sets are evenly assigned to the 9 runs and only some of them had to be used more than twice. In each run one data set of another light setting was reserved as validation data set, while using the other eight data sets for training. After nine runs of parameter estimation, the resulting mean parameter values were tested against one data set of each light setting that had not been used as training data before and was left over as a test data set. The description of the datasets used for training, as validation data and test data is given in Tables 17 and 18 in the Online Resource. The 9 runs of parameter estimation were each repeated 100 times, variating the starting values randomly out of a normal distribution with 3% relative standard deviation. So, a distribution of 900 values for all free parameters was obtained, the means and variation is given in Table 6.

**Table 6:** Mean values and coefficients of variation for model parameters $\mu _\mathrm{max}$, $\mu _\mathrm{d}$ and $K_\mathrm{I}$, based on the parameter estimations in all nine runs

Parameter	Mean value	Coefficient of variation [%]
$\mu _\mathrm{max}$ [1/h]	0.13	5.4
$\mu _\mathrm{d}$ [1/h]	0.0026	20
$K_\mathrm{I}$ [$\upmu$mol / (m² s)]	96	0.4

**Fig. 5:**
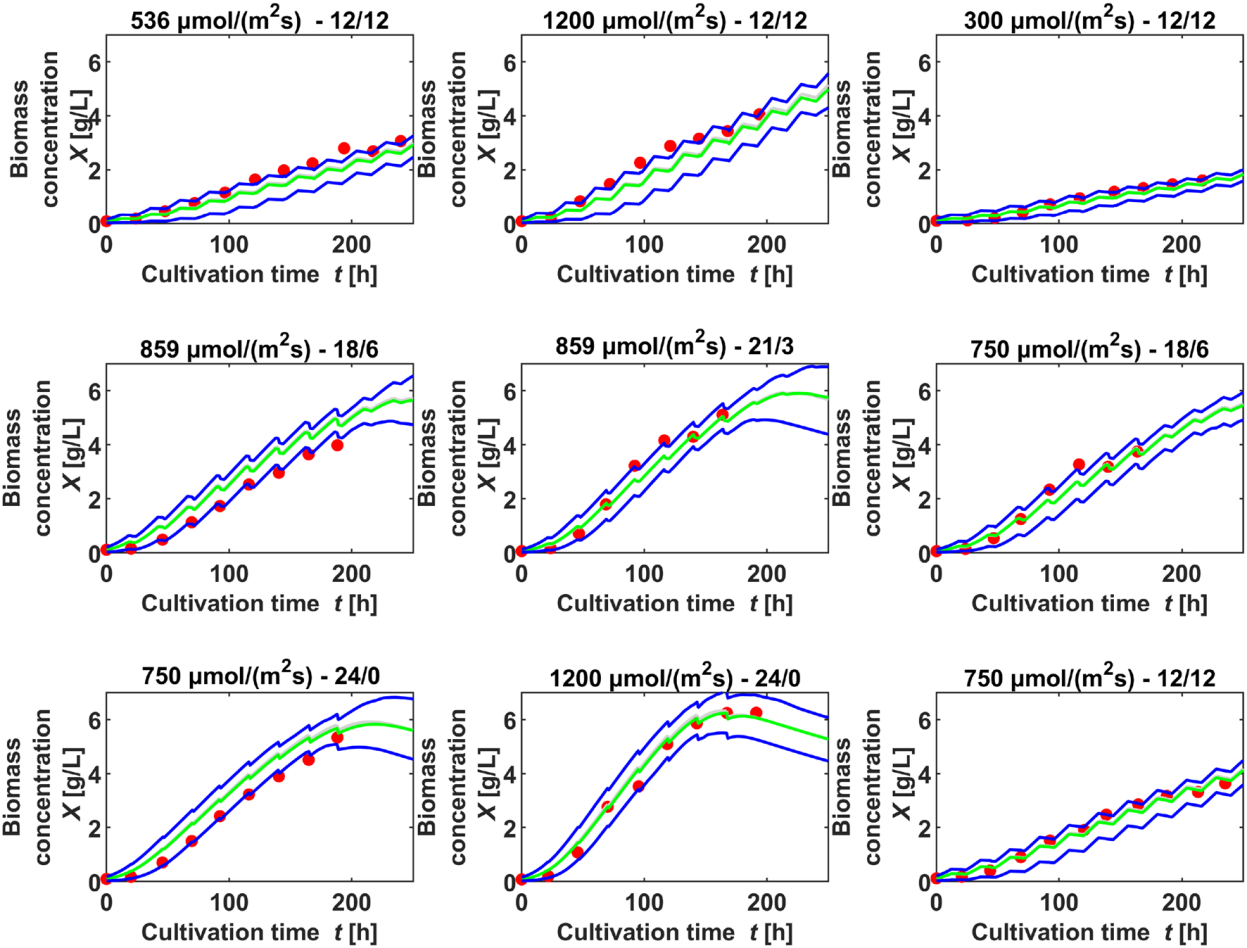
Example of the comparison of simulation (lines) and experimental data (dots) applying parameters sampled from the mean parameter distribution (dark lines: 5%- and 95%-quantiles simulation, pale lines: mean simulation)

The small coefficient of variation for $K_\mathrm{I}$ shows that the values for $K_\mathrm{I}$ all tend to the upper bound. To evaluate this, several parameter estimations were done setting the upper bound of $K_\mathrm{I}$ to higher values. In all cases, $K_\mathrm{I}$ tended to the higher bounds as well, in return the values for $\mu _\mathrm{max}$ rose to unreasonable values: (e.g. $K_\mathrm{I}$ = 192, $\mu _\mathrm{max}$ = 0.20). On the other hand, restriction of $K_\mathrm{I}$ to lower values resulted in lower estimations for $\mu _\mathrm{max}$. Because of this interdependency, either $K_\mathrm{I}$ or $\mu _\mathrm{max}$ had to be restricted in the upper bound, to estimate the other parameter freely. A value of 96 $\upmu$mol /(m$^2$ s) for $K_\mathrm{I}$ is in accordance with its meaning as half saturation coefficient for light: a maximal growth rate of 0.07 1/h was observed at values for $I_\mathrm{avg}$ of about 200 $\upmu$mol /(m$^2$ s). In addition, with $K_\mathrm{I}$ at much higher or much lower values, the consistency regarding simulations and validation data sets was lower. To evaluate the agreement between simulations and experimental data, for each dataset of the estimation 100 simulations with a randomly chosen parameter set from the estimations, variating the starting values randomly out of a normal distribution with 3% relative standard deviation, were used to compare the experimental biomass with the 90% prediction bands of the simulations. As an example for such a comparison, dataset run 2 is shown in Fig. 5.

Apart from the visualization in Fig. 5, the coefficients of determination for biomass simulation versus the respective validation data sets were calculated as in Tables 7 and 8.

**Table 7:** Coefficient of determination ($R^2$) of simulated and experimental biomass concentration for nine validation data sets at different light settings

Validation data sets			
Light setting: intensity [$\upmu$mol /(m$^2$ s)]	Duration [h]	Flask	$R^2$
536	12–12	5	0.815
1200	12–12	5	0.866
300	12–12	4	0.920
859	18–6	1	0.673
859	21–3	2	0.975
750	18–6	4	0.985
750	24–0	1	0.821
1200	24–0	5	0.870
750	12–12	5	0.989

**Table 8:** Coefficient of determination ($R^2$) and normalized root-mean-square deviation (NRMSE) of simulated and experimental biomass concentration for nine test data sets not used as training data

Test data sets				
Light setting: intensity [$\upmu$mol /(m$^2$ s)]	Duration [h]	Flask	$R^2$	NRMSE
536	12–12	1	0.761	0.165
1200	12–12	1	0.873	0.124
300	12–12	1	0.976	0.057
859	18–6	2	0.913	0.108
859	21–3	1	0.959	0.074
750	18–6	1	0.986	0.043
750	24–0	2	0.808	0.152
1200	24–0	1	0.940	0.090
750	12–12	1	0.953	0.077

**Fig. 6:**
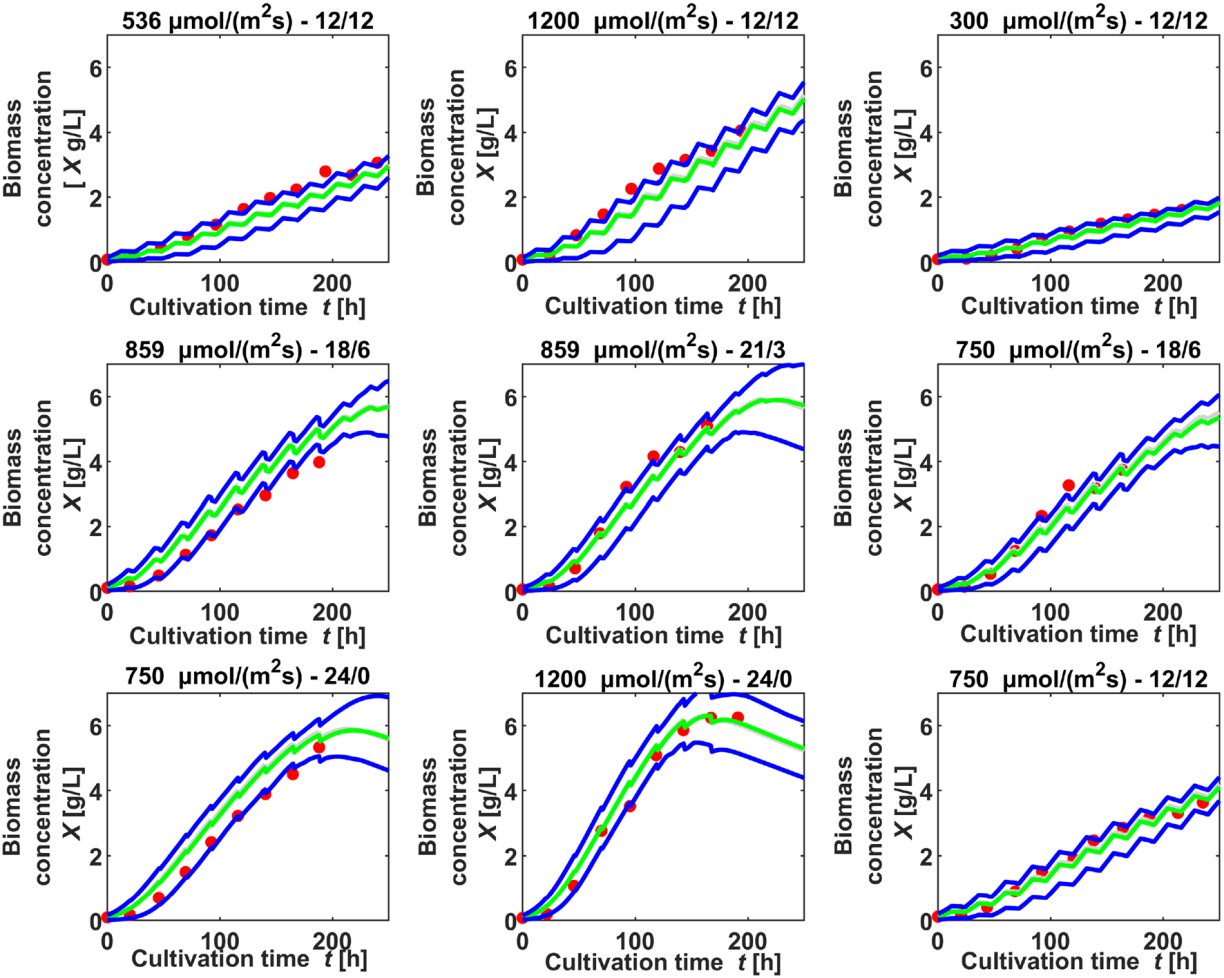
Comparison of simulation (lines) and experimental test data (dots) sampling from the mean parameter distribution for all settings (dark lines: 5%- and 95%-quantiles simulation, pale lines: mean simulation), the light intensity and light duration is given above each comparison

**Fig. 7:**
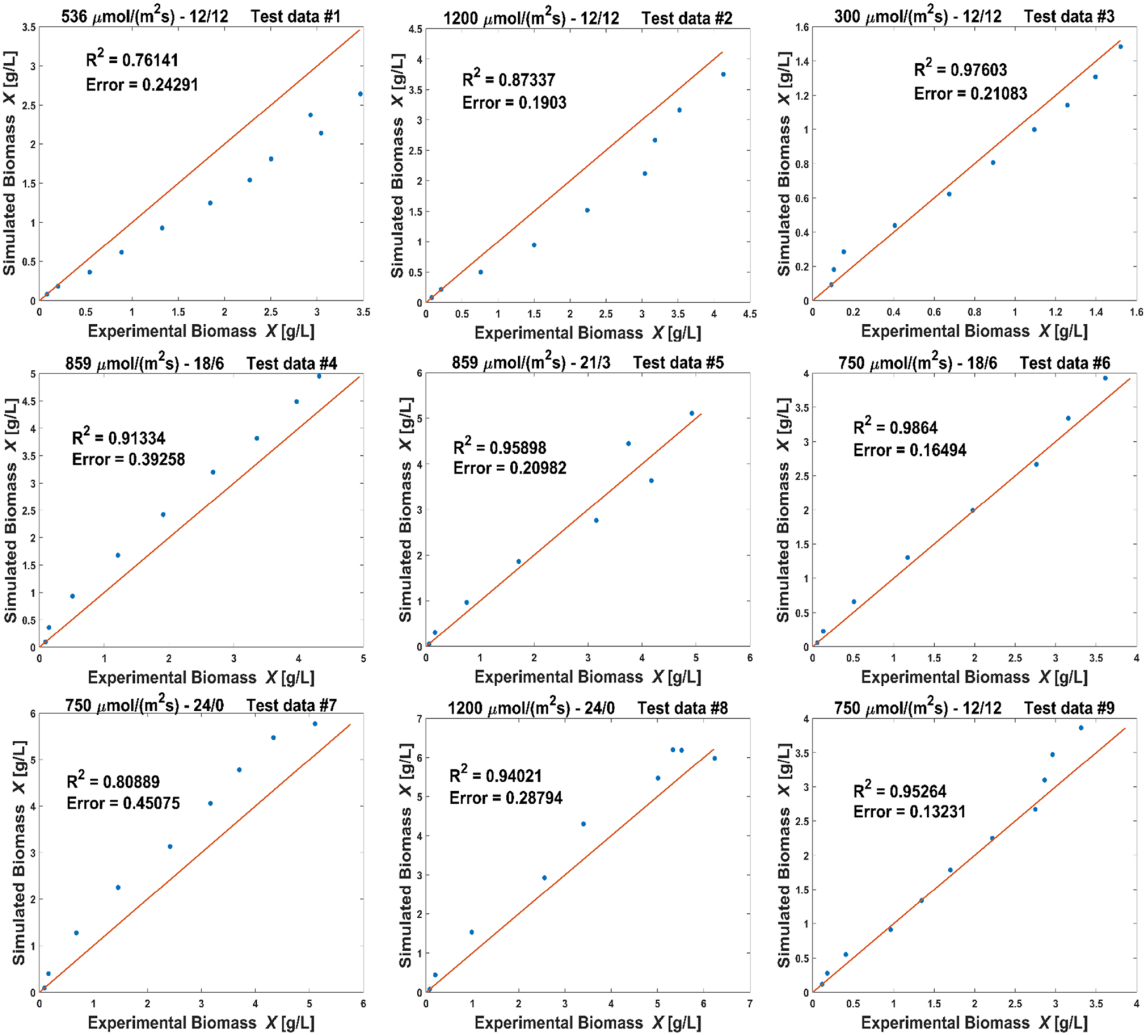
Comparison of simulated values, using the mean values of parameters as in Table 6, and experimental data (dots), and the diagonal that represents $R^2$ = 1 (line), above each comparison the light intensity and light duration of the applied test data set is specified

Whereas some of the validation data sets had been also used as training data sets in other estimations, nine data sets, one of every light setting, were completely left out of the training data, see Table 18 in the Online Resource. These data sets were each compared with a simulation of the same light setting and coefficients of determination are given in Table 8, along with the corresponding normalized root-mean-square deviations. Fig. 6 shows the simulated biomass compared with the measured biomass in the experiments conducted at 9 different light settings. Simulations were repeated 100 times, sampling initial values from a normal distribution with 3% variance and sampling parameters from a distribution of means of all estimations. The 5%- and 95% quantiles of these simulations are compared with experimental data in Fig. 6. The test data sets confirm the simulated values in a broad range of light intensities and durations, applying one parameter distribution in all simulations for different light settings. The goodness of fit is depicted as deviation from the diagonal in Fig. 7: The *y* values of the dots represent simulated values from the simulations using the mean estimated parameter distribution from all runs of the main parameter estimation. The *x* values of the dots are the values of the experimental test data not used in an estimation. If the dots lie below the diagonal line, the simulation underestimates the test data, if they lie above, the simulation overestimates. As Fig. 7 shows, all dots are near to the diagonal.

**Fig. 8:**
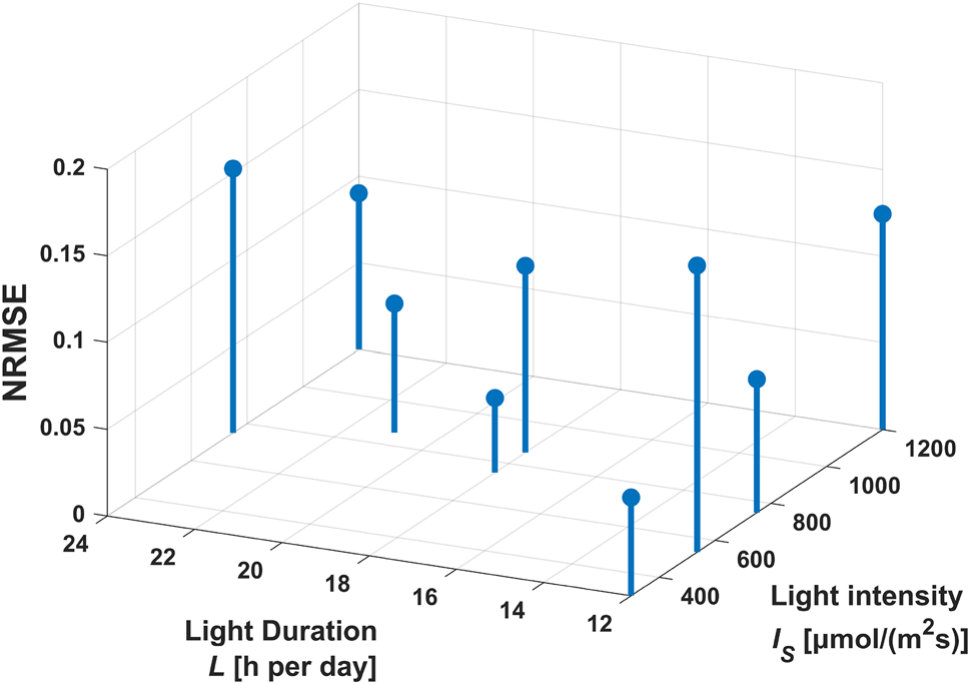
Representation of the normalized root-mean-square deviations (NRMSE) between the simulated and experimental biomass for different settings, ordered by light intensity and light duration

The normalized root-mean-square deviation for all settings is below 16% (Table 8). Taking into account that the variation between two cultivations of the same setting is up to 8% , for instance see Fig. 1, the model allows predictions for other settings in the ranges of the investigated light intensities and durations with similar precision. Some data sets are underestimated by the simulation, like 1 and 2, whereas some are overestimated, like 4 and 7. In order to inquire, if the deviation is dependent on light intensity or light duration, the normalized root-mean-square deviations (NRMSE) for all settings are plotted in Fig. 8 as a function of light intensity and light duration. The higher and lower values of NRMSE are randomly scattered in both directions, there was no correlation found between NRMSE and rising light intensity or duration. This suggests that the inaccuracies in the predicted biomass are random and independent from the light setting. That may be a sign that the equations can reproduce adequately the impact of light duration and light intensity on the growth rate. Although Zhang et al. [37] rate the prediction abilities of mechanistic models as poor, here the model, along with the estimated parameter set provided an applicable forecast on biomass growth in various light settings. This required, however, two conditions: The training data for parameter estimation must comprise several different settings of the evaluated variables and these settings should cover the ranges of the variables to be evaluated.

## Conclusion

A mechanistic model for biomass growth of *C. vulgaris* was adapted to the shake flask setting, using equations from Cornet et al. [29] for light attenuation and from del Rio-Chanona et al. [33] for nitrate consumption. 54 shake flask cultivations at different light conditions, regarding light hours per day and light intensity, were performed and the biomass data was used to conduct a stepwise parameter estimation: First exploring parameters concerning light and then focusing on the nitrate parameters, the growth parameters were identified in the main parameter estimation as third step. The availability of numerous cultivation data sets with individual combinations of light intensity and lighting duration allowed to estimate a set of mean parameter values suitable to simulate biomass growth for different light intensities and periods. Experimental versus simulated data was tested for nine different light settings, evaluating how far the model equations can predict biomass growth for variegated light intensities between 300 and 1200 $\upmu$mol /(m$^2$ s) and hours of light per day. As the deviations of predicted biomass from experimental data did not show to be dependent either of light intensity or of light period, the chosen equations can display the impact of light on *C. vulgaris* growth in wide ranges. Concordant with model simulations, high biomass concentrations in comparison with cultivations of *C. vulgaris* in literature were achieved by abundant lighting in shake flasks. So this contribution may be a helpful resource for modeling of phototrophic growth in the issue of describing light attenuation.

## Supplementary Information

Below is the link to the electronic supplementary material.

Supplementary file1 (PDF 549 KB)

## Abbreviations

EDTA: Ethylenediaminetetraacetic acid
NRMSE: Normalized root-mean-square deviations
OD: Optical density
PAR: Photosynthetically active radiation
pCO$_\mathrm{2}$: Dissolved carbon dioxide
PFD/PPFD: Photon flux density/photosynthetically active photon flux density
pO$_\mathrm{2}$: Dissolved oxygen
PPF: Photosynthetic photon flux
RPM: Revolutions per minute
SAG: Collection of Algae Cultures of the University of Göttingen
V/V: Volume per volume

## List of symbols

$\alpha$: Total extinction coefficient or linear scattering modulus
$\varepsilon$: Ratio spherical to hemispherical light per biomass concentration and height, m^2^/kg
$$\lambda$$: Wavelength, nm
$\mu$: Specific growth rate, 1/h
$\mu _D$: Specific death rate, 1/h
$\mu _\mathrm{max}$: Maximum specific growth rate, 1/h
*b*: Backward scattering fraction
$$c_\mathrm{N}$$: Nitrate concentration, g/L
$c_\mathrm{N,Feed}$: Nitrate concentration in the feed, g/L
$E_\mathrm{a}$: Mass absorption coefficient, m^2^/kg
$E_\mathrm{s}$: Mass scattering coefficient, m^2^/kg
$F_\mathrm{M}$: Medium feed rate, L/h
$$F_\mathrm{S}$$: Sample rate, L/h
*h*: Height, m
$I_\mathrm{avg}$: Average light intensity, μmol/(m^2^s)
$I_\mathrm{s}$: Set light intensity (light source), μmol/(m^2^s)
$I_0$: Incident light intensity, μmol/(m^2^s)
$K_\mathrm{I}$: Light associated Monod kinetic constant, μmol/(m^2^s)
$K_\mathrm{N}$: Nitrate uptake associated Monod kinetic constant, g/L
*L*: Light duration, h
$R^2$: Coefficient of determination
*t*: Time, h
*V*: Volume, L
*X*: Biomass concentration, (g/L)
$Y_\mathrm{N/X}$: Ratio of maximum nitrate uptake rate and maximum specific growth rate, $\mathrm g_{nitrate}/g_{biomass}$
*h * or *D*: Depth of the system, m
