# Correction note to: View on a mechanistic model of *Chlorella vulgaris* in incubated shake flasks

**DOI:** 10.1007/s00449-022-02687-y

**Published:** 2022-01-27

**Authors:** Fabian Kuhfuß, Veronika Gassenmeier, Sahar Deppe, George Ifrim, Tanja Hernández Rodríguez, Björn Frahm

**Affiliations:** 1 Biotechnology and Bioprocess Engineering, Ostwestfalen-Lippe University of Applied Sciences and Arts, Campusallee 12, Lemgo, Germany; 2 Frauenhofer IOSB-INA, Lemgo, Germany; 3 “Dunarea de Jos” University of Galati, Galati, Romania

## Bioprocess and Biosystems Engineering10.1007/s00449-021-02627-2

We noticed that the article contains the following errors, which have been corrected:

- In equation number 8a the variable *X* has been missing at the end. (This equation also appears in the graphical abstract. Article and graphical abstract have been updated.)
- The unit of biomass concentration *X* has been corrected in Table 2 and in the list of abbreviations (g/L instead of g/(L h)).
- A typo in the second affiliation has been corrected.
- The online resource document has been updated. (The previous document was not the final version.)

This has no impact on the conclusions of the article. The authors would like to apologize for any inconvenience caused.

