# Supplementary material for: View on a mechanistic model of Chlorella vulgaris in incubated shake flasks
Source: Bioprocess Biosyst Eng. 2021 Oct 22;45(1):15–30. doi: 10.1007/s00449-021-02627-2 (PMC8732984; doi:10.1007/s00449-021-02627-2)
Supplement: Supplementary file 1 — Supplementary file1 (PDF 549 KB) [file 449_2021_2627_MOESM1_ESM.pdf]

Online Supplement to: View on a mechanistic model of *C. vulgaris* in incubated shake flasks

Kuhfuß, Fabian [1*] ·
Gassenmeier, Veronika [1*] ·
Deppe, Sahar [2] · **Ifrim, George** [3] ·
Hernández Rodríguez, Tanja [1] ·
Frahm, Björn [1**]

[1] Ostwestfalen-Lippe University of Applied Sciences and Arts, Biotechnology & Bioprocess Engineering, Lemgo, Germany
[2] Fraunhofer IOSB - INA, Lemgo, Germany
[3] Dunarea de Jos University of Galati, Galati, Romania
*Equal first authorship
** Corresponding Author
Björn Frahm, Ostwestfalen-Lippe University of Applied Sciences and Arts, Biotechnology & Bioprocess Engineering, Campusallee 12, Lemgo
Phone: +49 5261 702-5663
E-mail: bjoern.frahm@th-owl.de

1 Medium Composition

Table 11: Composition of components for 1 L of Kessler medium

Composition	Mass [g] Standard medium	Mass [g] Modified medium
KNO_3	0.81	3.00
NaCl	0.47	0.47
$NaH_2PO_4 \cdot$ 2 H_2O	0.47	0.47
$Na_2HPO_4 \cdot$ 12 H_2O	0.36	0.36
$MgSO_4 \cdot$ 7 H_2O	0.25	0.4
$CaCl_2 \cdot$ 2 H_2O	0.014	0.014
$FeSO_4 \cdot$7 H_2O	0.006	0.006
$MnCl_2 \cdot$ 4 H_2O	0.0005	0.0005
H_3BO_3	0.0005	0.0005
$ZnSO_4 \cdot$ 7 H_2O	0.0002	0.0002
$(NH_4)_6Mo_7O_{24} \cdot$ 4 H_2O	0.00002	0.00002
Disodium-EDTA	0.008	0.008
H_2O (bidistilled)	1000	1000

2 Calculation of volume integral for average light intensity

To solve this equation, the investigated volume V has to be defined. Since the flasks are filled only in the lower space, to a height of 0.025 m, a cylindrical volume shape can be assumed for the cultivation system. This assumption may not fully represent the real shape of a shake flask but approximates well the current situation as depicted in Fig. 9. Therefore, cylindrical coordinates can be used to solve the integral, meaning that the integrated volume will be defined as $dV = d\varphi \, dr \, dz$.

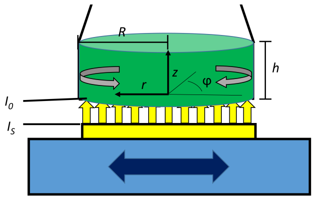

Fig. 9: Schematic diagram of the setting in the lighted and shaken flasks. I_S represents the set light intensity of the light source, I_0 is the light actually reaching the inside of the flask, r,φ and z are the coordinates of the working volume V, R and h are measures of the setting.

Based on these assumptions the mathematical description for the average light intensity can be noted as the following:

$$I_\text{avg} = \frac{1}{V} \cdot \int_0^{2\pi} \int_0^{R} \int_0^{h} I_0 \cdot e^{-(\varepsilon \cdot X \cdot z)} d\varphi \, dr \, dz \tag{1}$$

Any point of a cylinder is defined by its three coordinates. These are defined as r (radial axis), φ (angular axis) and z (upright coordinate). In this case r describes if a point is in the middle or the periphery, φ describes on which side in the cylinder a point is and z describes at which height a point is. As the lighting in the investigated setting is located under the flask in upright direction and evenly distributed over the whole surface, it can be assumed that the incident light is equal for all cells in the same level above the ground. So, the incident light on the shaken cells in the flask is independent of φ and r, it only depends on z, leading to

$$I_\text{avg} = \frac{1}{V} \cdot \int_0^{h} I_0 \cdot e^{-(\varepsilon \cdot X \cdot z)} \, dz \; = \; I_0 \cdot \frac{1}{h \cdot \varepsilon \cdot X} \cdot (1 - e^{-(\varepsilon \cdot X \cdot h)}) \tag{2}$$

3 Development of pH over Cultivation time

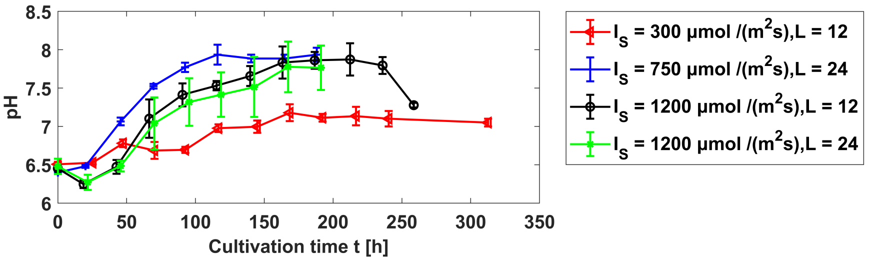

Fig. 10: Diagram of the measured pH values over cultivation time showing that pH values stay within 6 to 8 in all performed cultivations, error bars describe the variations between different flasks of one setting

4 Datasets for Training, Validation and Test

4.1 Light parameter estimation

Table 12: Settings of light intensity, different height in the flask and biomass concentrations for the measurements of local incident light that were used in the light parameter estimation:

Light intensities I_S:	300 µmol/(m^{2}s), 750 µmol/(m^{2}s), 1200 µmol/(m^{2}s)
Heights in the flask h:	0.1 cm, 0.5 cm, 1 cm, 1.5 cm, 2 cm
Biomass concentrations X:	0.2 g/L, 0.5 g/L, 1 g/L, 1.5 g/L, 2 g/L, 3 g/L, 4 g/L

4.2 Nitrate Parameter Estimation

Table 13: Initial values and bounds for parameter estimation including nitrate data

Parameter	Unit	Initial value (S)	Lower bound	Upper bound
μ_{max}	[1/h]	0.08	0.3·S	2·S
μ_d	[1/h]	0.001	0.5·S	2·S
K_I	[m^2/kg]	64	0.5·S	1.5·S
K_N	[g/L]	0.14	0.5·S	1.5·S
Y_{NX}	[mg/mg]	0.15	0.5 ·S	1.5·S

Table 14: Light settings of nitrate data sets

	536 µmol/(m^{2}s), 12-12			1200 µmol/(m^{2}s), 12-12		
Flask Number	1	2	3	4	5	6

Table 15: Classification of nitrate data sets into training- validation- and test data

Parameter Estimation Nitrate					
Distribution of Flask Numbers 1 to 6					
Run	Validation data	Training data			
Run 1	6	2	3	4	5
Run 2	5	2	3	4	6
Run 3	4	2	3	5	6
Run 4	3	2	4	5	6
Run 5	2	3	4	5	6
Test data	1				

4.3 Main Estimation

Table 16: Initial values and bounds for the main parameter estimation

Parameter	Unit	Status	Initial value (S)	Lower bound	Upper bound
μ_{max}	[1/h]	free	0.14	0.5·S	1.5·S
μ_{d}	[1/h]	free	0.002	0.5·S	1.5·S
K_{I}	[m^2/kg]	free	64	0.5·S	1.5·S
K_{N}	[g/L]	fix	0.15		
Y_{NX}	[mg/mg]	fix	0.24		
E_{a}	[m^2]	fix	227		
E_{s}	[m^2/kg]	fix	800		
b		fix	0.0008		

Table 17: Light settings of data sets used for the main estimation

No. of data set	Light intensity [µmol/(m^2s)]	Hours light-dark [h]
1	536	12-12
2	1200	12-12
3	300	12-12
4	859	18-6
5	859	21-3
6	750	18-6
7	750	24-0
8	1200	24-0
9	750	12-12

Table 18: Assignment of the flasks to training- validating- or test data in the main estimation

Run:	No. of data set and no. of flask (F)<br>Validation data:	Training data:
rn1	1 F5	2 F2, 3 F6, 4 F1, 5 F3, 6 F3, 7 F1, 8 F12, 9 F12
rn2	2 F5	1 F6, 3 F5, 4 F1, 5 F2, 6 F2, 7 F1, 8 F11, 9 F11
rn3	3 F4	1 F4, 2 F4, 4 F1, 5 F4, 6 F4, 7 F1, 8 F10, 9 F10
rn4	4 F1	1 F3, 2 F3, 3 F3, 5 F3, 6 F5, 7 F1, 8 F9, 9 F9
rn5	5 F2	1 F2, 2 F6, 3 F2, 4 F1, 6 F6, 7 F1, 8 F8, 9 F8
rn6	6 F4	1 F2, 2 F2, 3 F2, 4 F1, 5 F4, 7 F1, 8 F7, 9 F7
rn7	7 F1	1 F6, 2 F6, 3 F6, 4 F1, 5 F3, 6 F3, 8 F6, 9 F6
rn8	8 F5	1 F5, 2 F5, 3 F5, 4 F1, 5 F2, 6 F2, 7 F1, 9 F5
rn9	9 F4	1 F4, 2 F4, 3 F4, 4 F1, 5 F4, 6 F4, 7 F1, 8 F4
Test data<br>1 F1, 2 F1, 3 F1, 4 F2, 5 F1, 6 F1, 7 F2, 8 F1, 9 F1		
